# Endoplasmic reticulum and Golgi apparatus are the preferential sites of Foscan® localisation in cultured tumour cells

**DOI:** 10.1038/sj.bjc.6600664

**Published:** 2003-01-28

**Authors:** M-H Teiten, L Bezdetnaya, P Morlière, R Santus, F Guillemin

**Affiliations:** 1 Unité de Recherche en Thérapie Photodynamique, Centre Alexis Vautrin, F-54511 Vandoeuvre-les-Nancy Cedex, France; 2 Laboratoire de Photobiologie, Muséum National d'Histoire Naturelle, Paris, France; 3 Institut de Recherche sur la Peau, INSERM U.532, Hôpital Saint-Louis, Paris, France

**Keywords:** Foscan®, intracellular localisation, microspectrofluorometry, confocal microscopy

## Abstract

Intracellular photosensitiser localisation significantly influences the mechanism of response to photodynamic therapy (PDT), since the primary sites of damage are closely related to the specific sensitiser distribution. Foscan® subcellular localisation in the MCF-7 human adenocarcinoma cell line has been studied by means of confocal microscopy and microspectrofluorometry. The fluorescence topographic profiles recorded after cells costained with Foscan® and organelle-specific fluorescent probes revealed that Foscan® presents low localisation in lysosomes and a weak accumulation in mitochondria. Alternatively, the Foscan® fluorescence topographic profile turned out to colocalise perfectly with that obtained for the endoplasmic reticulum (ER) and the Golgi apparatus. The patterns of fluorescence derived from confocal microscopy studies were consistent with predominant localisation of Foscan® in these organelles. Furthermore, evaluation of enzymatic activity of selected organelles immediately after laser light irradiation (650 nm) indicated the Golgi apparatus and ER as the primary damaged sites resulting from Foscan®-mediated PDT in the MCF-7 cell line. To our knowledge, this is the first study to demonstrate unambiguously that the ER and the Golgi apparatus are preferential sites of Foscan® accumulation.

Photodynamic therapy (PDT) is a local treatment used for the eradication of light-accessible solid tumours. This therapy is based upon the systemic or local administration of photosensitising compound followed by illumination with visible or near-infrared light (Henderson and Dougherty, 1992). The photosensitiser absorbs light and in the presence of oxygen transfers energy, producing short-lived cytotoxic oxygen species such as singlet oxygen or other oxygen radicals (Sharman *et al*, 2000). The ultimate phototoxic effect of photosensitisers is governed by their photophysical properties as well as their intratumoural and intracellular uptake with subsequent translocation to various membrane-delimited compartments. Since singlet oxygen, the main cytotoxic species produced by PDT, is not able to diffuse over distances longer than 10–20 nm during its excited state lifetime and cell dimensions are approximately 20 *μ*m, the primary sites of photodynamic action should be strictly related to the specific intracellular sensitiser distribution (Moan and Berg, 1991). Still, the correlation between the sites of specific sensitiser localisation and primary light-induced damage sites should be established for every given photosensitiser.

*Meta*-tetra(hydroxyphenyl)chlorin (*m*THPC, Foscan®) is a second-generation photosensitiser (Bonnett *et al*, 1989) that appears to be one of the most effective sensitisers studied to date (Dougherty *et al*, 1998). Recently, a regulatory approval was granted for the Foscan®-PDT palliative treatment of head and neck cancers. Clinical trials for other neoplastic diseases are currently in an advanced stage (Ris *et al*, 1996; Baas *et al*, 2001; Wyss *et al*, 2001). Despite numerous studies on Foscan®'s mechanisms of action, an enigma still persists regarding the reason(s) for the high photodynamic efficiency of this sensitiser. Its photophysical properties, such as quantum yields of triplet state and singlet oxygen generation (Bonnett *et al*, 1999), are comparable or even inferior to other clinically relevant photosensitisers (Blum and Grossweiner, 1985; Aveline *et al*, 1994). Another factor determining the effective outcome of Foscan® photosensitisation could be its intracellular localisation. Two recent studies from our group, performed using fluorescence microscopy, addressed the intracellular localisation of Foscan® in HT29 and MCF-7 cultured cells (Melnikova *et al*, 1999; Teiten *et al*, 2001). In both cell lines we noted diffuse intracellular distribution in the cytoplasm outside the nucleus with intense fluorescence in the perinuclear region, where the nuclear membrane and the Golgi/endoplasmic reticulum (ER) complex are located. Our observations were consistent with those from other groups (Ma *et al*, 1994; Hornung *et al*, 1997; Yow *et al*, 2000). At present, no preferential subcellular target of Foscan® has been identified.

The microspectrofluorometry technique allows the investigation of fluorescence in real time in localised cellular compartments (Santus *et al*, 1991; Morlière *et al*, 1998; Matroule *et al*, 1999). Spectral and topographic information available from areas smaller than 1 *μ*m^2^ make it possible to characterise fairly specific sites of localisation and to follow the chronology of the photosensitised reactions induced by specific probes.

Bearing this in mind, we performed a detailed analysis of Foscan® *in vitro* subcellular distribution in MCF-7 human breast adenocarcinoma cell line by means of microspectrofluorometry and confocal laser scanning microscopy. We further attempted to identify the primary photoinactivation sites through the study of the enzymatic activities of selected organelles.

## MATERIALS AND METHODS

### Chemicals

Foscan® was kindly supplied by Biolitec Pharma Ltd (Edinburgh, UK). Foscan® stock solution was prepared in methanol. Further dilution was performed in phenol-red-free RPMI 1640 medium (Life Technologies, Cergy-Pontoise, France) supplemented with 2% fetal calf serum (FCS, Dutscher, Brumath, France) to reach a final concentration of 1 *μ*g ml^−1^ (1.5 *μ*M) Foscan®. The fluorescence probes used for staining the Golgi apparatus (*N*-(4,4-difluoro-5,7-dimethyl-4-bora-3a,4a-diaza-*s*-indacene-3-pentanoyl) sphingosine, BODIPY® FL C_5_-ceramide, BPC) and the ER (3,3′-dihexyloxacarbocyanine iodide, DiOC_6_) were purchased from Molecular Probes-Europe (Leiden, The Netherlands). The probes used for staining mitochondria (rhodamine 123, Rh123) and lysosomes (lucifer yellow, LY) were provided by Sigma (Sigma-Aldrich, France).

### Treatment of cells prior to microspectrofluorometry and confocal laser scanning microscopy

The MCF-7 human breast adenocarcinoma cell line was cultivated in phenol-red-free RPMI 1640 medium supplemented with 9% FCS, penicillin (10 000 IU) and streptomycin (10 000 IU). Exponentially growing MCF-7 cells (1×10^4^ cells ml^−1^) were plated in 2 ml of culture medium as monolayers in 35-mm Nunc Petri dishes containing a microscope slide for microspectrofluorometric analysis or in chambered coverglass (Nunc, Polylabo) for confocal laser scanning microscopy (CLSM) observations. Cells were incubated with 1 *μ*g ml^−1^ Foscan® for 3 h, and unless otherwise indicated, washed with phosphate-buffered saline (PBS) and incubated with a series of fluorescent probes to determine the identity of subcellular organelles targeted by Foscan®. For lysosomes identification, cells were incubated overnight with 125 *μ*g ml^−1^ LY, washed and incubated for 3 h with 1 *μ*g ml^−1^ Foscan® (Haugland, 1996).

Rh123 was used at a final concentration of 10 *μ*M for 45 min to identify mitochondria with microspectrofluorometry and at a concentration of 5 *μ*M for 30 min, with CLSM (Kim *et al*, 1998). These concentration and incubation conditions are optimal for the specific staining of mitochondria in carcinoma-derived cell lines, including MCF-7 (Nadakavukaren *et al*, 1985). To visualise the Golgi apparatus, cells were labelled with 4 *μ*M BPC for 15 min (Pagano *et al*, 1991). The ER was labeled with the lipophilic, cationic DiOC_6_ dye applied for 15 min at a final concentration of 2 *μ*g ml^−1^ (Terasaki *et al*, 1984). This marker, used in the concentration range 1–10 *μ*g ml^−1^, was reported to be highly specific for ER and, compared to other possible candidates, is brightest with the slowest bleaching rate (Sabnis *et al*, 1997). At the end of the double staining, the labelling solution was removed by gentle rinsing with RPMI 1640 containing 25 mM Hepes, and microspectrofluorometry measurements or observations by confocal fluorescence microscopy were performed.

### Confocal laser scanning microscopy

The cells double stained with Foscan® and organelle probes were examined with a confocal laser scanning microscope (ASP-2 AOBS CLSM, Leica microsystem, Germany) equipped with a ×63, numerical aperture 1.3 oil immersion objective (Leica, Germany). A pinhole of 60.85 *μ*m was used and each image recorded contained 512×512 pixels. An He/Ne laser was used as excitation light at 458 nm for all organelle probes and at 633 nm for Foscan®. Fluorescence of the organelles probes was detected on channel 1 with a 505–545 nm band pass (BP) emission filter. Channel 2 was used to detect the red fluorescence of Foscan® with a 640–660 nm BP emission filter. The fluorescence images were displayed in green and red ‘false’ colour output and electronically combined to visualise colocalisation in yellow. Controls (cells stained only with Foscan® or organelle probes) were conducted in parallel to optimise the staining protocol and the detecting parameters of CLSM.

### Microspectrofluorometric techniques

The microspectrofluorometer used is centred around a Leitz ‘Diavert’ inverted microscope and has been described previously (Morlière *et al*, 1998). Fluorescence was excited over the whole microscopic field through the objective lens (×40, ×63 and ×100) with 435 nm light from an OSRAM HBO 100 W mercury lamp. A slit in the primary image plane, which delineates a 1.5 *μ*m area in the microscopic field from which the fluorescence can be collected, is used as an entrance slit of a 150 grooves mm^−1^ grating and provides 5 nm spectral resolution. Fluorescence photons received by a two-dimensional, cooled charge-coupled device target produce a ‘spectrotopographic image’. Such images can be interpreted as either a succession in the *X*-direction of linear, monochromatic images of a 1.5 *μ*m wide strip or a succession of spectra along the *Y*-axis, each corresponding to an area 1.5×0.21 *μ*m (×40 objective), 1.5×0.135 *μ*m (×63 objective) or 1.5×0.085 *μ*m (×100 objective) of the strip.

### Enzymatic activity measurements

Logarithmically growing MCF-7 cells were incubated with 1 *μ*g ml^−1^ Foscan® over 3 h. At the end of the incubation period, cells were washed three times, reincubated in fresh culture medium and irradiated with a 650 nm laser diode light (Coherent, France) at a fluence rate of 2.12 mW cm^−2^ for different light doses. Immediately after irradiation, cytotoxicity was determined by clonogenic assay as previously described (Merlin *et al*, 1992). Colonies composed of more than 50 cells were counted 14 days later with an automatic image analysis program (AnalySiS 3.1). The effect of PDT on the activity of enzymes located in different cellular compartments was evaluated immediately after 180 s of light exposure corresponding to 0.74 J cm^−2^. At this light fluence, 99% of the cells were inhibited from forming colonies (lethal dose 99%, LD_99_).

For the determination of cytochrome *c* oxidase activity, the mitochondria were isolated from cells. The enzymatic activity was then measured as described by Gibson and Hilf (1983) by a spectrophotometric technique recording the disappearance of cytochrome *c*_red_ (reduced form of the substrate cytochrome *c* (Sigma) at 550 nm).

The UDP galactosyl transferase activity was measured from extracts of microsomes as described by Brandli *et al* (1988). This method is based on the ability of this enzyme to bind the radioactive-labelled uridine diphospho-D-galactose (Amersham, France), to ovalbumin, which can be monitored by scintillation counting of the radioactivity with a Beckman automatic liquid scintillation system.

The NADPH cytochrome *c* reductase activity was measured from extracts of microsomes as described by Beaufay *et al* (1974). This method is based on the formation of the reduced form of cytochrome *c*, cytochrome *c*_red_, induced by NADPH, which can be recorded spectrophotochemically at 550 nm.

## RESULTS

### Intracellular Foscan® localisation assessed by confocal laser scanning microscopy

**Figure 1:**
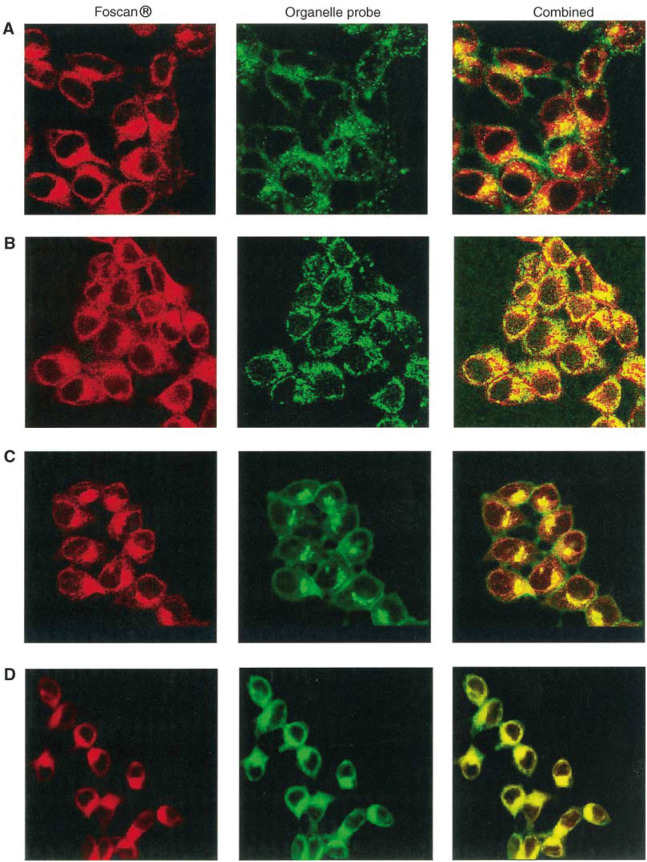
Confocal fluorescence images of MCF-7 cells double stained with Foscan® and organelle probes. Lysosomes were stained by overnight incubation with 125 *μ*g ml^−1^ LY, washed and incubated for 3 h with 1 *μ*g ml^−1^ Foscan® (row A). For other organelle probes, cells were sensitised with Foscan® (1 *μ*g ml^−1^, 3 h), washed and then subjected to organelle staining. Mitochondria were labeled with 5 *μ*M Rh123 for 30 min (row B), Golgi apparatus was stained with 4 *μ*M BPC (15 min) (row C) and ER with 2 *μ*g ml^−1^ DiOC_6_ for 15 min (row D). Objective magnification×63.

Confocal micrographs of the double-stained MCF-7 cells with Foscan® and the organelle markers are presented in Figure 1

. The representative images of Foscan® are shown in red (left panel), the organelle-specific dyes in green (middle panel), and the overlapped images in yellow (right panel).

Foscan® presents an intracellular fluorescence distribution in cytoplasmic compartments with no obvious fluorescence in the nucleus (Figure 1). Subcellular localisation was further examined using costaining with organelle probes. Figure 1 (row a) shows that the staining patterns of lysosomal probe LY and Foscan® were different, thus indicating that very little Foscan® accumulated inside the lysosomes. The mitochondrial image showed a partial overlap with the corresponding Foscan® image (Figure 1, row b).

The Golgi probe BPC requires special attention. Two wavelengths of emission should be considered for this probe. Incorporation of BPC in the Golgi apparatus and in the Golgi vesicles promotes the aggregation of the probe, which emits a red fluorescence at 621 nm. In the monomeric form, it binds to intracellular membranes (e.g., the ER, nuclear envelope) and emits a green fluorescence at 545 nm. However, by detecting green fluorescence, it was found that this dye accumulates also in a region of intense green fluorescence identified as the Golgi body (Pagano *et al*, 1991). Considering the red fluorescence of Foscan®, only a green fluorescence filter could be used to observe BPC in the micrographs shown in Figure 1 (row C). A good superposition between the Golgi dye image and that of Foscan® was evident. This was expected since both dyes, being lipophilic, have a preference for the membranous organelles of the MCF-7 cells. The bright fluorescence in the perinuclear region was presumed to be a Golgi body. The presence of BPC aggregates will be addressed in the topographic spectra analysis of Foscan® intracellular localisation.

A clear colocalisation of Foscan® and DiOC_6_ in the ER was observed (Figure 1, row D).

### Intracellular Foscan® localisation assessed by microspectrofluorometry

Figure 2

**Figure 2:**
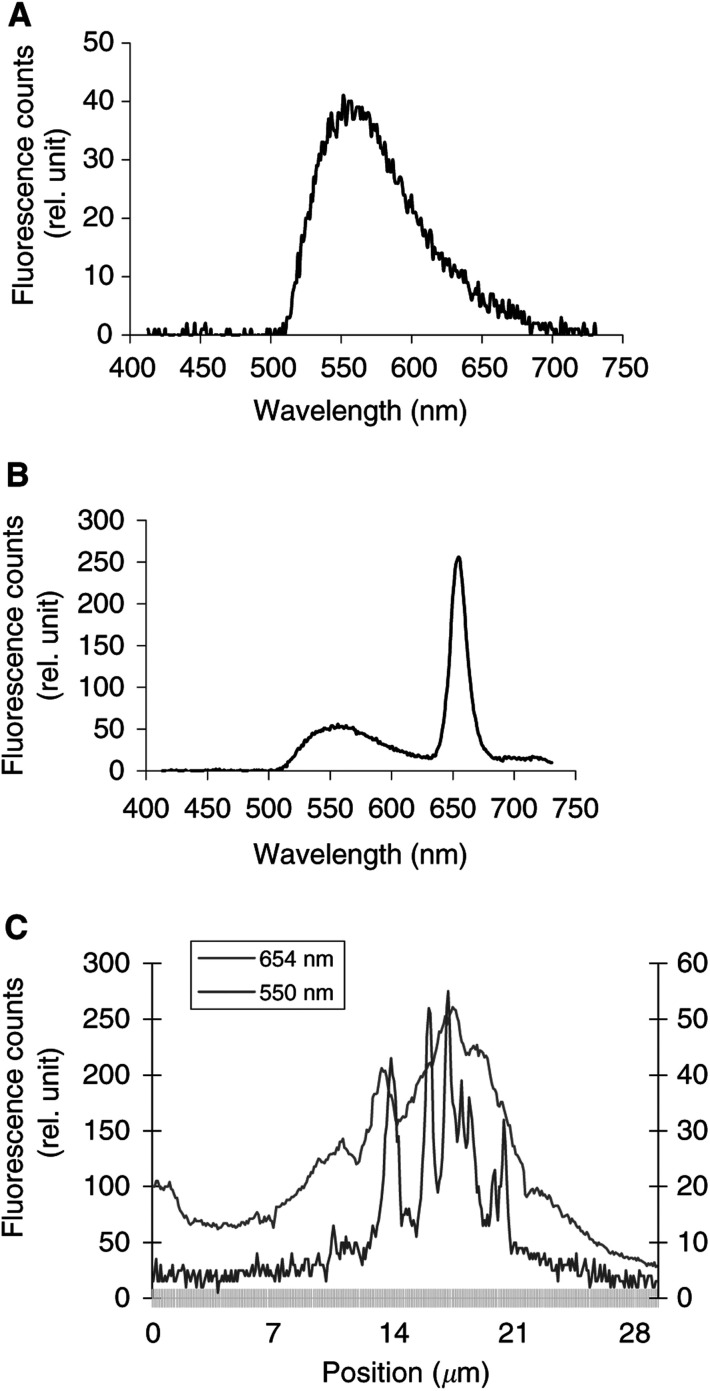
Intracellular fluorescence spectra of LY alone (**A**). Intracellular fluorescence spectra (**B**) and fluorescence topographic profiles (**C**) of MCF-7 cells incubated overnight with 125 *μ*g ml^−1^ LY and Foscan® (1 *μ*g ml^−1^, 3 h). The spectrum in **(B)** was recorded at position 16.6 *μ*m on the *X*-axis of the profile in (**C**). Fluorescence topographic profiles in (**C**) were recorded at 550 nm (LY) and at 654 nm (Foscan®). Excitation wavelength 435 nm, objective magnification×40, exposure time 4 s.

, 3

**Figure 3:**
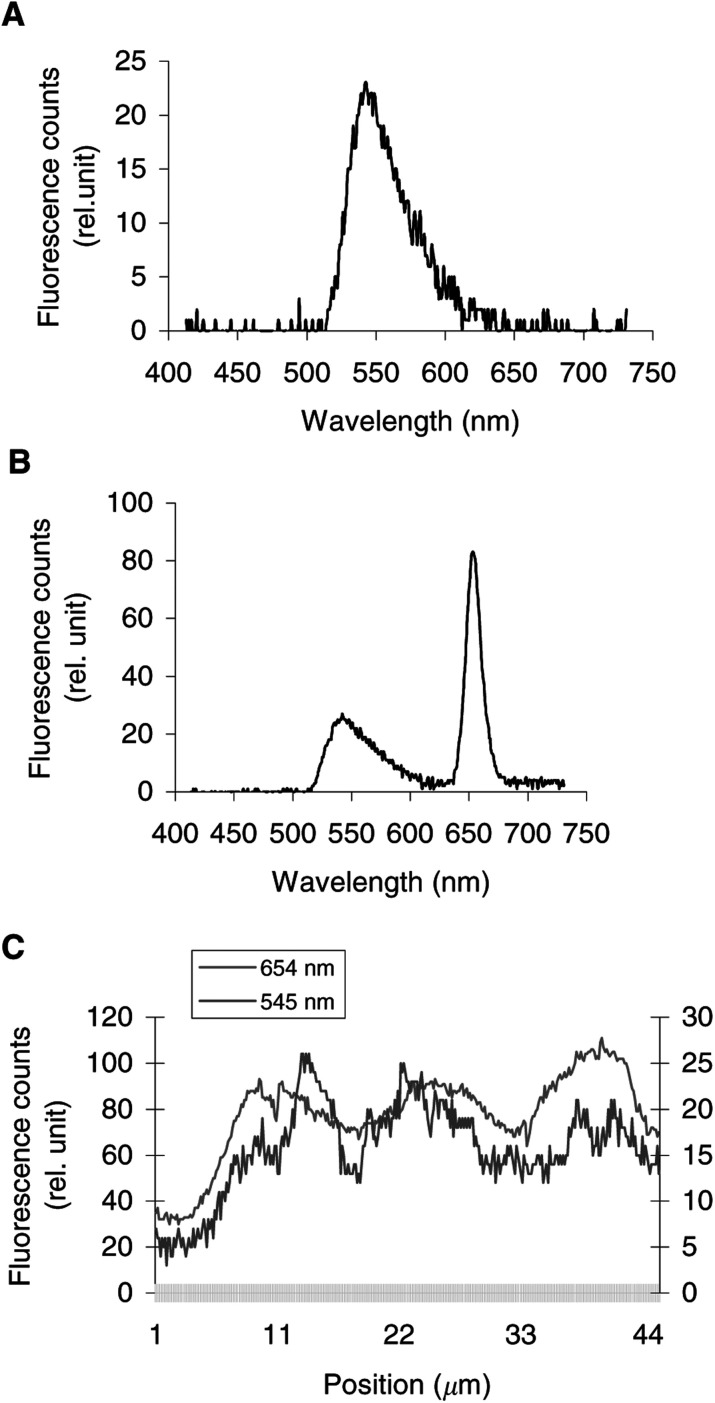
Intracellular fluorescence spectra of Rh123 alone (**A**). Intracellular fluorescence spectra (**B**) and fluorescence topographic profiles (**C**) of MCF-7 cells incubated for 3 h with 1 *μ*g ml^−1^ Foscan® and then with 10 *μ*M Rh123 for 45 min. The spectrum in (**B**) was recorded at position 13.5 *μ*m on the *X*-axis of the profile in (**C**). Fluorescence topographic profiles in (**C**) were recorded at 545 nm (Rh123) and at 654 nm (Foscan®). Excitation wavelength 435 nm, objective magnification×63, exposure time 4 s.

, 4

**Figure 4:**
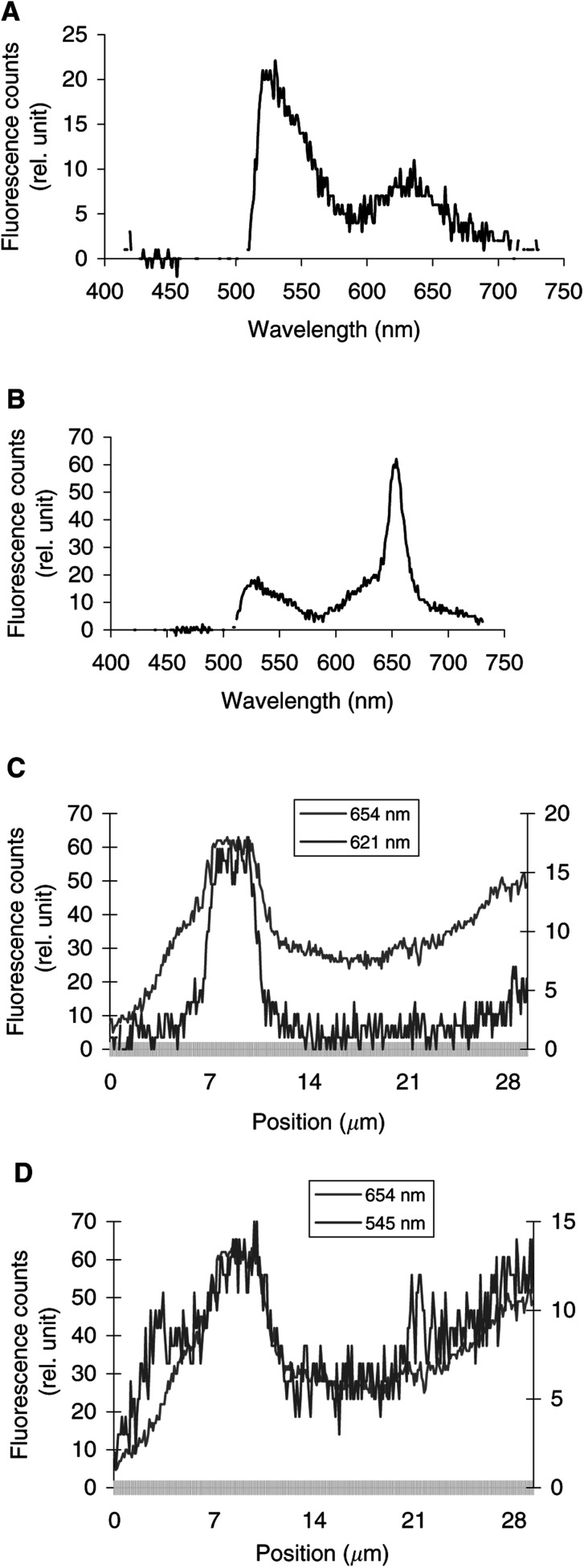
Intracellular fluorescence spectra of BPC alone (**A**). Intracellular fluorescence spectra (**B**) and fluorescence topographic profiles (**C**) of MCF-7 cells incubated for 3 h with 1 *μ*g ml^−1^ Foscan® and then with 4 *μ*M BPC during 15 min. The spectrum in (**B**) was recorded at position 8.5 *μ*m on the *X*-axis of the profiles in (**C**) and (**D**). Fluorescence topographic profiles of Foscan® (654 nm) and BPC, recorded either at 621 nm, which corresponds to the Golgi apparatus (**C**), or at 545 nm, which corresponds to intracellular membranes (**D**). Excitation wavelength 435 nm, objective magnification×100, exposure time 4 s.

and 5

**Figure 5 fig5:**
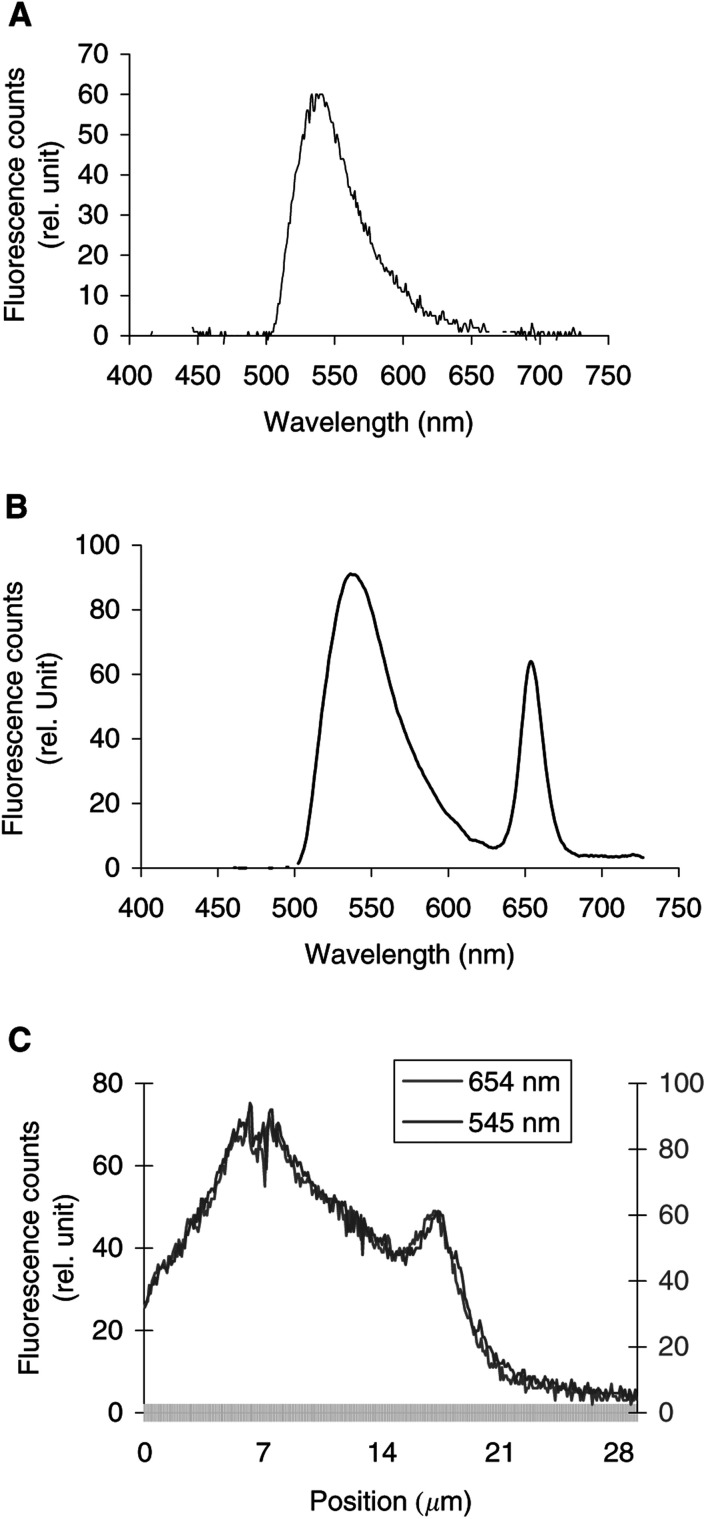
Intracellular fluorescence spectra of DiOC_6_ alone (**A**). Intracellular fluorescence spectra (**B**) and fluorescence topographic profiles (**C**) of MCF-7 cells incubated for 3 h with 1 *μ*g ml^−1^ Foscan® and then with 2 *μ*g ml^−1^ DiOC_6_ for 15 min. The spectrum in (**B**) was recorded at position 5.70 *μ*m on the *X*-axis of the profile in (**C**). Fluorescence topographic profiles in (**C**) were recorded at 545 nm (DiOC_6_) and at 654 nm (Foscan®). Excitation wavelength 435 nm, objective magnification×100, exposure time 4 s.

display the fluorescence spectra of the organelle markers alone (panels a), the fluorescence spectra of Foscan® and the organelle markers (panels b), and the topographic profiles of both compounds (panels c and d) from the single living cell. The topographic profiles were recorded along a strip within the cell at the specific emission wavelengths of Foscan® and organelle probes. It should be noted that the fluorescence emission of all organelle probes was well separated from that of Foscan® (*λ*_em_=654 nm).

Results of measurements performed after coincubation of cells with Lucifer Yellow (LY), are presented in Figure 2. The fluorescence topographic profile of Foscan® did not match that obtained with LY (*λ*_em_=550 nm) as the spikes of LY fluorescence at lysosomic locations did not overlap the red profile of Foscan® (Figure 2c). In other words, both lysosomes and cellular compartments in the close vicinity that are devoid of lysosomes were stained by Foscan® in a similar mode.

Figure 3c shows the fluorescence topographic profiles of Foscan® and Rh123 (*λ*_em_=545 nm). Both profiles exhibit quite a similar tendency without the extensive overlap between them.

To evaluate the possible localisation of Foscan® in the Golgi apparatus/ER complex, Foscan®-loaded cells were incubated with BPC. Figure 4a shows that the fluorescence spectrum of BPC in MCF-7 cells has two maxima, at 545 and 621 nm. The red fluorescence of BPC indicates aggregates formation in the perinuclear areas, which correspond to Golgi apparatus (Pagano *et al*, 1991). The fluorescence topographic profile of BPC, recorded at 621 nm, shows a good superposition with that of Foscan®, thus indicating their good colocalisation (Figure 4C). Even better colocalisation was observed when BPC fluorescence was recorded at 545 nm (Figure 4D). In order to determine whether this latter colocalisation pattern corresponds to Foscan® accumulation in the ER, cells were costained with DiOC_6_ (*λ*_em_=545 nm). The plots of Figure 5 demonstrate that the Foscan® fluorescence topographic profile turned out to colocalise perfectly with that obtained for DiOC_6_. These data unambiguously indicate a good localisation of Foscan® in the ER and in the Golgi apparatus of MCF-7 cells.

### Foscan® sensitised photoinactivation of intracellular enzymes

Despite the direct correlation between the sites of dye location and sites of direct photodamage, the probability always exists that a low level of incorporation into a given compartment may give rise to a high impact of photochemical reaction. Therefore, in the next step we addressed the immediate post-PDT damage of enzymes known as markers for subcellular organelles.

The activity of the mitochondrial enzyme cytochrome *c* oxidase was apparently not affected by Foscan® photosensitisation, since its activity was fully preserved in cells undergoing 99% death. Under the same irradiation conditions, the enzymatic activity of NADPH cytochrome *c* reductase associated with the ER was reduced by 54.0±6.3%, while that of the UDP galactosyl transferase located in the Golgi apparatus was reduced by 78±5.25%.

## DISCUSSION

The main goal of the present study was the identification of the specific subcellular localisation of Foscan®, as it yields valuable information about the primary targets of Foscan®-PDT. Correlating the sites of dye location and sites of direct photodamage seems allowable since photochemically produced singlet oxygen is expected to diffuse intracellularly to distances limited by its short lifetime to 10–20 nm (Moan and Berg, 1991).

Lysosomes have been shown to be a potential site of localisation for aggregated and/or hydrophilic sensitizers (Gèze *et al*, 1993; Berg and Moan, 1997). These organelles have been implicated in a cell death mechanism mediated through the photoinduced production of ceramide (Sawai and Hannun, 1999). The photomicrographs (Figure 1) and the fluorescence topographic profiles (Figure 2) recorded in cells double stained with Foscan® and LY do not favour Foscan® accumulation in lysosomes.

There is strong evidence that sensitisers with an acute localisation in mitochondria promote the release of cytochrome *c* upon irradiation (Xue *et al*, 2001). This loss of cytochrome *c* can be lethal to cells either because of the disruption of the mitochondrial respiratory chain with the eventual reduction of cellular ATP levels or through caspase initiation with subsequent apoptotic cell death (Yow *et al*, 2000; Xue *et al*, 2001). Figure 3C shows that mitochondrial-localising Rh123 and Foscan® fluorescence topographic patterns were different, indicating a poor affinity of Foscan® for these organelles. This observation was consistent with the photomicrograph of dual labeling of Foscan® and Rh123 (Figure 1, row B).

The ER and the Golgi apparatus are closely linked not only by their location in the perinuclear area of the cytoplasm, but also as they interact together in the case of newly synthesised proteins. The ER is known to play a central role in the biosynthesis, segregation and transport of proteins and lipids as well as in the release of intracellular stores of calcium (Terasaki *et al*, 1984). The Golgi apparatus receives newly synthesised proteins from the ER and modifies them chemically, for example, by glycosylation or sulphonation (Short and Barr, 2000). The impact of PDT on the ER and Golgi apparatus has not often been taken into account. Only a few studies have demonstrated the importance of photochemical damage to the Golgi apparatus and ER for the inactivation of tumour cells in culture (Morlière *et al*, 1998; Matroule *et al*, 1999; Fabris *et al*, 2001). Thus, photochemical damage to the ER has been proposed to explain the exceptional effectiveness of tolyporphin compared to other sensitisers (Morlière *et al*, 1998). Recently, the Golgi apparatus was found to be a primary site of photodamage in cells photosensitised with zinc (II) phthalocyanine (Fabris *et al*, 2001).

From the photomicrographs of cells costained with Foscan® and BPC (Figure 1C), it appears rather difficult to deduce whether Foscan® localises in the Golgi complex, as the green fluorescence of BPC is not specific for the Golgi body. The use of microspectrofluorometry makes possible the identification of the Golgi apparatus since the red fluorescence of the Golgi probe could be well separated from that of Foscan® (Figure 4). The comparison of Foscan® and BPC topographic profiles presented in Figure 4C, D demonstrated a good localisation of Foscan® in the Golgi apparatus and even better colocalisation when BPC's green fluorescence was registered, pointing out the staining of different intracellular membranes including that of the ER. Foscan®'s localisation in the ER was further unambiguously confirmed by the Foscan®/DiOC_6_ double staining assessed by confocal microscopy and microspectrofluorometry (Figure 1 and 5). In particular, Figure 5C demonstrates a perfect colocalisation of Foscan® and DiOC_6_ fluorescence topographic profiles.

Photosensitised damage to enzymes characteristic of subcellular organelles gives information about the intracellular localisation of the sensitiser and could be indicative of the primary photodamage sites (Rodal *et al*, 1998; Fabris *et al*, 2001). The measurements of the enzymatic activities of selected organelles after Foscan® photosensitisation reinforce the conclusion of the predominant localisation of Foscan® in the ER and Golgi apparatus. The lack of inactivation of the mitochondrial marker enzyme cytochrome *c* oxidase in cells undergoing almost complete photokilling indicates low mitochondrial localisation of Foscan®. This observation does not exclude the possibility that Foscan® molecules accumulated in mitochondrial sites remote from the marker enzyme investigated and toward which singlet oxygen could not easily diffuse. Meantime, the activities of NADPH cytochrome *c* reductase, which localises in the ER, and UDP galactosyl transferase, a marker enzyme for the Golgi apparatus, were significantly decreased in cells immediately after Foscan®-based PDT. It is worth noting that Foscan® localisation in the endoplasmic reticulum led to only partial (54%) inhibition of NADPH cytochrome *c* reductase activity, whereas much better correlation appears between the extent of inhibition of UDP galactosyl transferase activity and Foscan® photosensitised cell inactivation. Further experiments are required before any detailed explanation can be evoked.

In conclusion, the present study, which is based primarily on the microspectrofluorometric analysis of cells costained with Foscan® and organelle-specific probes, has clearly shown that Foscan® exhibits low or no localisation in lysosomes and only a weak accumulation in mitochondria. It has been unambiguously demonstrated that the Golgi apparatus and ER are preferential sites of Foscan® accumulation. To our knowledge, this is the first study to verify Foscan® localisation in these organelles. Furthermore, measurements of subcellular marker enzyme photoinactivation may indicate the Golgi apparatus and ER as the primary therapy-induced damage sites resulting from Foscan®-mediated PDT in the MCF-7 cell line. The final identification of the intracellular sites crucial for photoinactivation could be done through establishing the causal relation between the damage brought to certain organelles and overall cytotoxicity.

## References

[bib1] Aveline B, Hasan T, Redmond RW (1994) Photophysical and photosensitizing properties of benzoporphyrin derivative monoacid ring A (BPD-MA). Photochem Photobiol 59: 328–335 8016212 10.1111/j.1751-1097.1994.tb05042.x

[bib2] Baas P, Saarnak AE, Oppelaar H, Neering H, Stewart FA (2001) Photodynamic therapy with *meta*-tetrahydroxyphenylchlorin for basal cell carcinoma: a phase I/II study. Br J Dermatol 145: 75–78 11453910 10.1046/j.1365-2133.2001.04284.x

[bib3] Beaufay H, Amar-Costesec A, Feytmans E, Thines-Sempoux D, Wibo M, Robbi M, Berthet J (1974) Analytical study of microsomes and isolated subcellular membranes from rat liver. I. Biochemical methods. J Cell Biol 61: 188–200 4150488 10.1083/jcb.61.1.188 PMC2109252

[bib4] Berg K, Moan J (1997) Lysosomes and microtubules as targets for photochemotherapy of cancer. Photochem Photobiol 65: 403–409 9077120 10.1111/j.1751-1097.1997.tb08578.x

[bib5] Blum A, Grossweiner LI (1985) Singlet oxygen generation by hematoporphyrin IX, uroporphyrin I and hematoporphyrin derivative at 546 nm in phosphate buffer and in the presence of egg phosphatidylcholine liposomes. Photochem Photobiol 41: 27–32 3157197 10.1111/j.1751-1097.1985.tb03443.x

[bib6] Bonnett R, White RD, Winfield UJ, Berenbaum MC (1989) Hydroporphyrins of the meso-tetra(hydroxyphenyl)porphyrin series as tumour photosensitizers. Biochem J 261: 277–280 2775215 10.1042/bj2610277 PMC1138813

[bib7] Bonnett R, Charlesworth P, Djelal BD, Foley S, McGarvey DJ, Truscott TG (1999) Photophysical properties of 5,10,15,20-tetrakis(*m*-hydroxyphenyl)-porphyrin (m-THPP) and 5,10,15,20-tetrakis(*m*-hydroxyphenyl)bacterio-chlorin (*m*-THPBC): a comparative study. J Chem Soc Perkin Trans 2: 325–328

[bib8] Brandli AW, Hansson GC, Rodriguez-Boulan E, Simons K (1988) A polarized epithelial cell mutant deficient in translocation of UDP-galactose into the Golgi complex. J Biol Chem 263: 16283–16290 3141404

[bib9] Dougherty TJ, Gomer CJ, Henderson BW, Jori G, Kessel D, Korbelik M, Moan J, Peng Q (1998) Photodynamic therapy. J Natl Cancer Inst 90: 889–905 9637138 10.1093/jnci/90.12.889 PMC4592754

[bib10] Fabris C, Valduga G, Miotto G, Borsetto L, Jori G, Garbisa S, Reddi E (2001) Photosensitization with zinc (II) phthalocyanine as a switch in the decision between apoptosis and necrosis. Cancer Res 61: 7495–7500 11606385

[bib11] Gèze M, Morlière P, Mazière JC, Smith KM, Santus R (1993) Lysosomes, a key target of hydrophobic photosensitizers proposed for photochemotherapeutic applications. J Photochem Photobiol B 20: 23–35 8229466 10.1016/1011-1344(93)80128-v

[bib12] Gibson SL, Hilf R (1983) Photosensitization of mitochondrial cytochrome *c* oxidase by hematoporphyrin derivative and related porphyries *in vitro* and *in vivo*. Cancer Res 43, 4191–4197 6307505

[bib13] Haugland RP (1996) Cell-permeant probes for lysosomes and other organic organelles. In: Handbook of Fluorescent Probes and Research Chemicals. N.T.Z. Spence (ed) Eugene, OR. Molecular Probes, Inc.

[bib14] Henderson BW, Dougherty TJ (1992) How does photodynamic therapy work? Photochem Photobiol 55: 145–157 1603846 10.1111/j.1751-1097.1992.tb04222.x

[bib15] Hornung R, Jentsch B, Crompton NE, Haller U, Walt H (1997) *In vitro* effects and localisation of the photosensitizers *m*-THPC and *m*-THPC MD on carcinoma cells of the human breast (MCF-7) and Chinese hamster fibroblasts (V-79). Lasers Surg Med 20: 443–450 9142685 10.1002/(sici)1096-9101(1997)20:4<443::aid-lsm11>3.0.co;2-c

[bib16] Kim M, Cooper DD, Hayes SF, Spangrude GJ (1998) Rhodamine-123 staining in hematopoietic stem cells of young mice indicates mitochondrial activation rather than dye efflux. Blood 91: 4106–4117 9596656

[bib17] Ma L, Moan J, Berg K (1994) Evaluation of a new photosensitizer, meso-tetra-hydroxyphenyl-chlorin, for use in photodynamic therapy: a comparison of its photobiological properties with those of two other photosensitizers. Int J Cancer 57: 883–888 8206681 10.1002/ijc.2910570618

[bib18] Matroule JY, Bonizzi G, Morlière P, Paillous N, Santus R, Bours V, Piette J (1999) Pyropheophorbide-a methyl ester-mediated photosensitization activates transcription factor NF-kappaB through the interleukin-1 receptor-dependent signaling pathway. J Biol Chem 274: 2988–3000 9915837 10.1074/jbc.274.5.2988

[bib19] Melnikova VO, Bezdetnaya LN, Bour C, Festor E, Gramain MP, Merlin JL, Potapenko, A, Guillemin F (1999) Subcellular localisation of *meta*-tetra (hydroxyphenyl) chlorin in human tumor cells subjected to photodynamic treatment. J Photochem Photobiol B 49: 96–103 10392459 10.1016/s1011-1344(99)00033-0

[bib20] Merlin JL, Azzi S, Lignon D, Ramacci C, Zeghari N, Guillemin F (1992) MTT assays allow quick and reliable measurement of the response of human tumour cells to photodynamic therapy. Eur J Cancer 28A: 1452–1458 1387543 10.1016/0959-8049(92)90542-a

[bib21] Moan J, Berg K (1991) The photodegradation of porphyrins in cells can be used to estimate the lifetime of singlet oxygen. Photochem Photobiol 53: 549–553 1830395 10.1111/j.1751-1097.1991.tb03669.x

[bib22] Morlière P, Mazière JC, Santus R, Smith CD, Prinsep MR, Stobbe CC, Fenning MC, Golberg JL, Chapman JD (1998) Tolyporphin: a natural product from cyanobacteria with potent photosensitizing activity against tumor cells *in vitro* and *in vivo*. Cancer Res 58: 3571–3578 9721863

[bib23] Nadakavukaren K, Nadakavukaren J, Chen LB (1985) Increased rhodamine 123 uptake by carcinoma cells. Cancer Res 45: 6093–6099 4063967

[bib24] Pagano RE, Martin OC, Kang HC, Haugland RP (1991) A novel fluorescent ceramide analogue for studying membrane traffic in animal cells: accumulation at the Golgi apparatus results in altered spectral properties of the sphingolipid precursor. J Cell Biol 113: 1267–1279 2045412 10.1083/jcb.113.6.1267 PMC2289039

[bib25] Ris HB, Altermatt HJ, Nachbur B, Stewart CM, Wang Q, Lim CK, Bonnett R, Althaus U (1996) Intraoperative photodynamic therapy with *m*-tetrahydroxyphenylchlorin for chest malignancies. Lasers Surg Med 18: 39–45 8850464 10.1002/(SICI)1096-9101(1996)18:1<39::AID-LSM5>3.0.CO;2-S

[bib26] Rodal GH, Rodal SK, Moan J, Berg K (1998) Liposome-bound Zn (II)-phthalocyanine. Mechanisms for cellular uptake and photosensitization. J Photochem Photobiol B 45: 150–159 9868805 10.1016/s1011-1344(98)00175-4

[bib27] Sabnis RW, Deligeorgiev TG, Jachak M N, Dalvi TS (1997) DiOC_6_ (3): a useful dye for staining the endoplasmic reticulum. Biotech Histochem 72: 253–258 9408585 10.3109/10520299709082249

[bib28] Santus R, Morlière P, Kohen E (1991) The photobiology of the living cell as studied by microspectrofluorometric techniques. Photochem Photobiol 54: 1071–1077 1837930 10.1111/j.1751-1097.1991.tb02131.x

[bib29] Sawai H, Hannun YA (1999) Ceramide and sphingomyelinases in the regulation of stress responses. Chem Phys Lipids 102: 141–147 11001568 10.1016/s0009-3084(99)00082-1

[bib30] Sharman WM, Allen CM, van Lier JE (2000) Role of activated oxygen species in photodynamic therapy. Methods Enzymol 319: 376–400 10907528 10.1016/s0076-6879(00)19037-8

[bib31] Short B, Barr FA (2000) The Golgi apparatus. Curr Biol 10: R583–R585 10985372 10.1016/s0960-9822(00)00644-8

[bib32] Teiten MH, Bezdetnaya L, Merlin JL, Bour-Dill C, Pauly ME, Dicato M, Guillemin F (2001) Effect of *meta*-tetra(hydroxyphenyl)chlorin (*m*THPC)-mediated photodynamic therapy on sensitive and multidrug-resistant human breast cancer cells. J Photochem Photobiol B 62: 146–152 11566278 10.1016/s1011-1344(01)00178-6

[bib33] Terasaki M, Song J, Wong JR, Weiss MJ, Chen LB (1984) localisation of endoplasmic reticulum in living and glutaraldehyde-fixed cells with fluorescent dyes. Cell 38: 101–108 6432338 10.1016/0092-8674(84)90530-0

[bib34] Wyss P, Schwarz V, Dobler-Girdziunaite D, Hornung R, Walt H, Degen A, Fehr M (2001) Photodynamic therapy of locoregional breast cancer recurrences using a chlorin-type photosensitizer. Int J Cancer 93: 720–724 11477585 10.1002/ijc.1400

[bib35] Xue LY, Chiu SM, Oleinick NL (2001) Photodynamic therapy-induced death of MCF-7 human breast cancer cells: a role for caspase-3 in the late steps of apoptosis but not for the critical lethal event. Exp Cell Res 263: 145–155 11161713 10.1006/excr.2000.5108

[bib36] Yow CM, Chen JY, Mak NK, Cheung NH, Leung AW (2000) Cellular uptake, subcellular localisation and photodamaging effect of temoporfin (*m*THPC) in nasopharyngeal carcinoma cells: comparison with hematoporphyrin derivative. Cancer Lett 157: 123–113 10.1016/s0304-3835(00)00453-5 10936672

